# Supplementary material for: SARS‐CoV‐2–host proteome interactions for antiviral drug discovery
Source: Mol Syst Biol. 2021 Oct 28;17(11):e10396. doi: 10.15252/msb.202110396 (PMC8552907; doi:10.15252/msb.202110396)
Supplement: Supplementary file 2 — Appendix [file MSB-17-e10396-s010.pdf]

Appendix
Table of content

Appendix Figure S1. Expression of the bait proteins in HEK293 cells.

Appendix Figure S2. Comparison of the gene expression profiles of stable cell lines.

Appendix Figure S3. Human receptor protein-protein interaction map.

Appendix Figure S4. Enriched Reactome pathways of prey proteins of the virus-host interactome.

Appendix Figure S5. Enriched Pfam terms of prey proteins of the virus-host interactome.

Appendix Figure S6. Enriched GO biological process terms of prey proteins of the virus-host interactome.

Appendix Figure S7. Enriched Reactome pathways of prey proteins of the host receptor interactome.

Appendix Figure S8. Enriched GO biological process terms of prey proteins of the host receptor interactome.

Appendix Figure S9. Enriched Pfam terms of prey proteins of the host receptor interactome.

Appendix Figure S10. Distribution of frequencies of hub proteins.

Appendix Figure S11. The antiviral activity of proposed repurposing candidates in the image-based drug screening of SARS-CoV-2.

Appendix Figure S12. The presence of BMS-863233 does not affect interactions formed with GLO1.

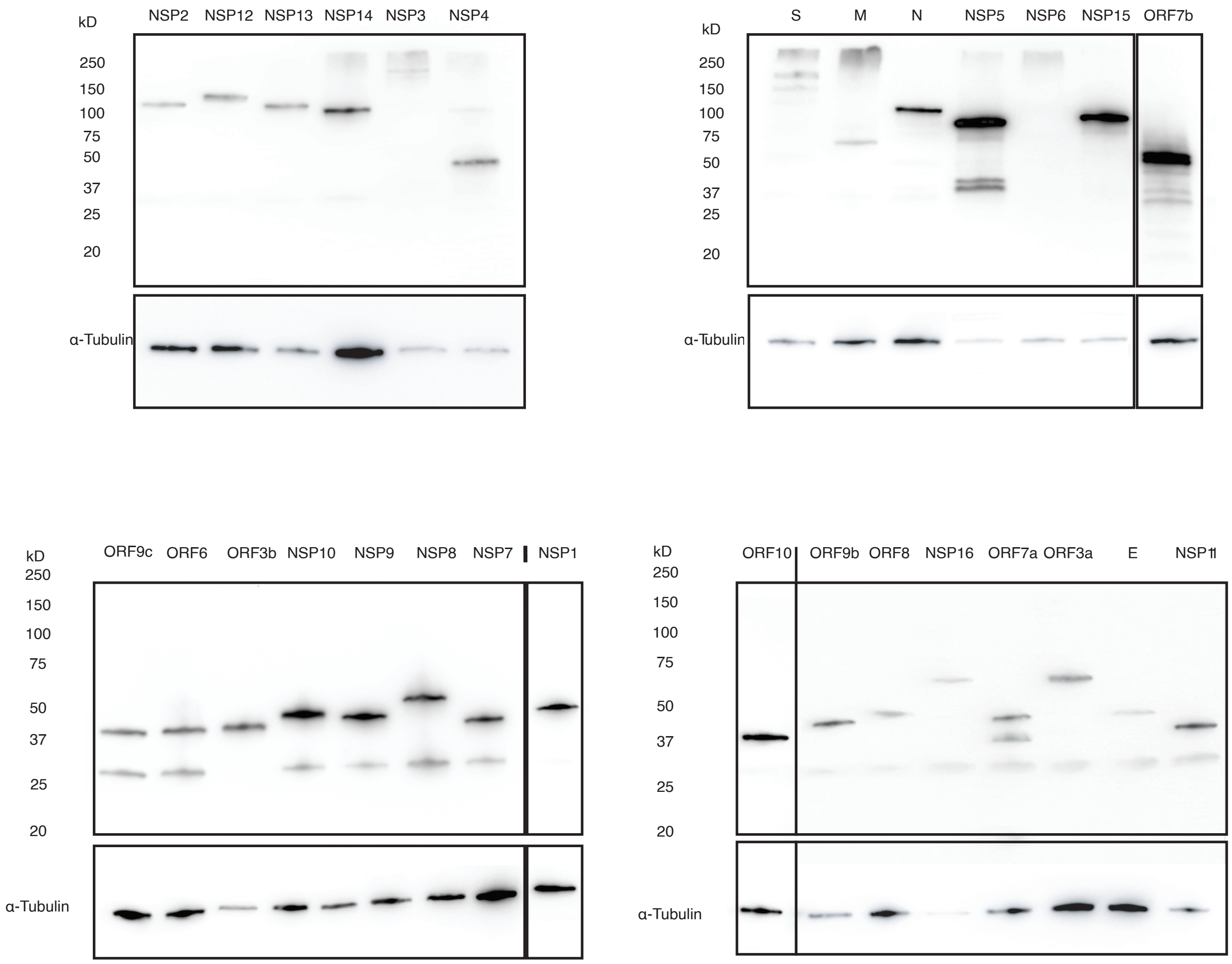

Appendix Figure S1. Expression of the bait proteins in HEK293 cells.
MAC-tagged bait protein expression in stable cell lines was detected by western blotting.
Bait proteins were blotted with an anti-HA antibody, and tubulin was used as the loading control

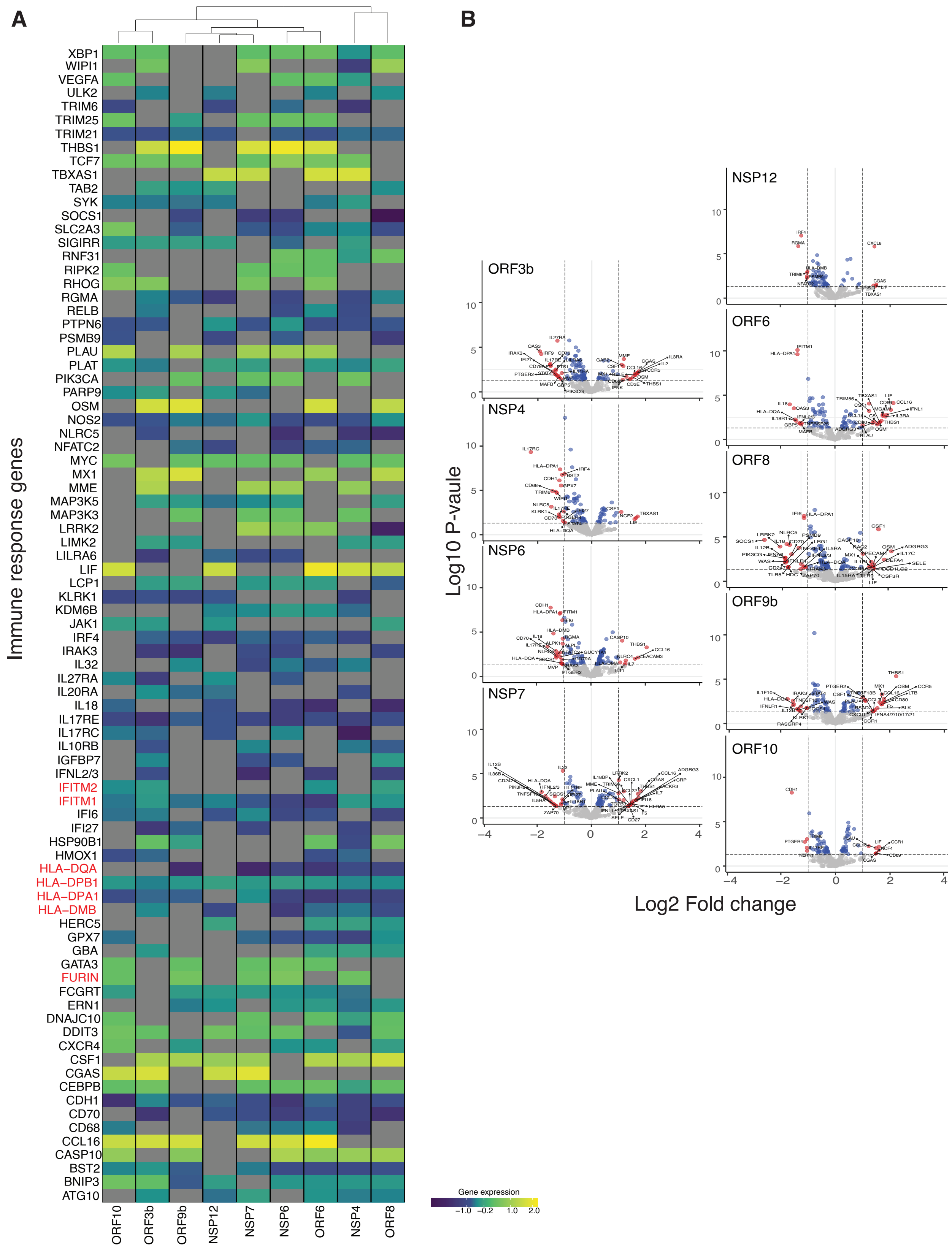

Appendix Figure S2. Comparison of the gene expression profiles of stable cell lines. (A)Heatmap of hierarchical clustering showing the expression profiles of immune response related genes in different viral ORF cell lines. Genes with normalized expression levels that were significant compared to those in the parental cell line (untransfected cell line as control) were present in at least four samples.

(B) Volcano plots produced from the NanoString gene expression data of stable cell lines. The red dots labeled with gene names represent those considered significantly differentially expressed (fold change > 2 (up or down) and P-value < 0.01).

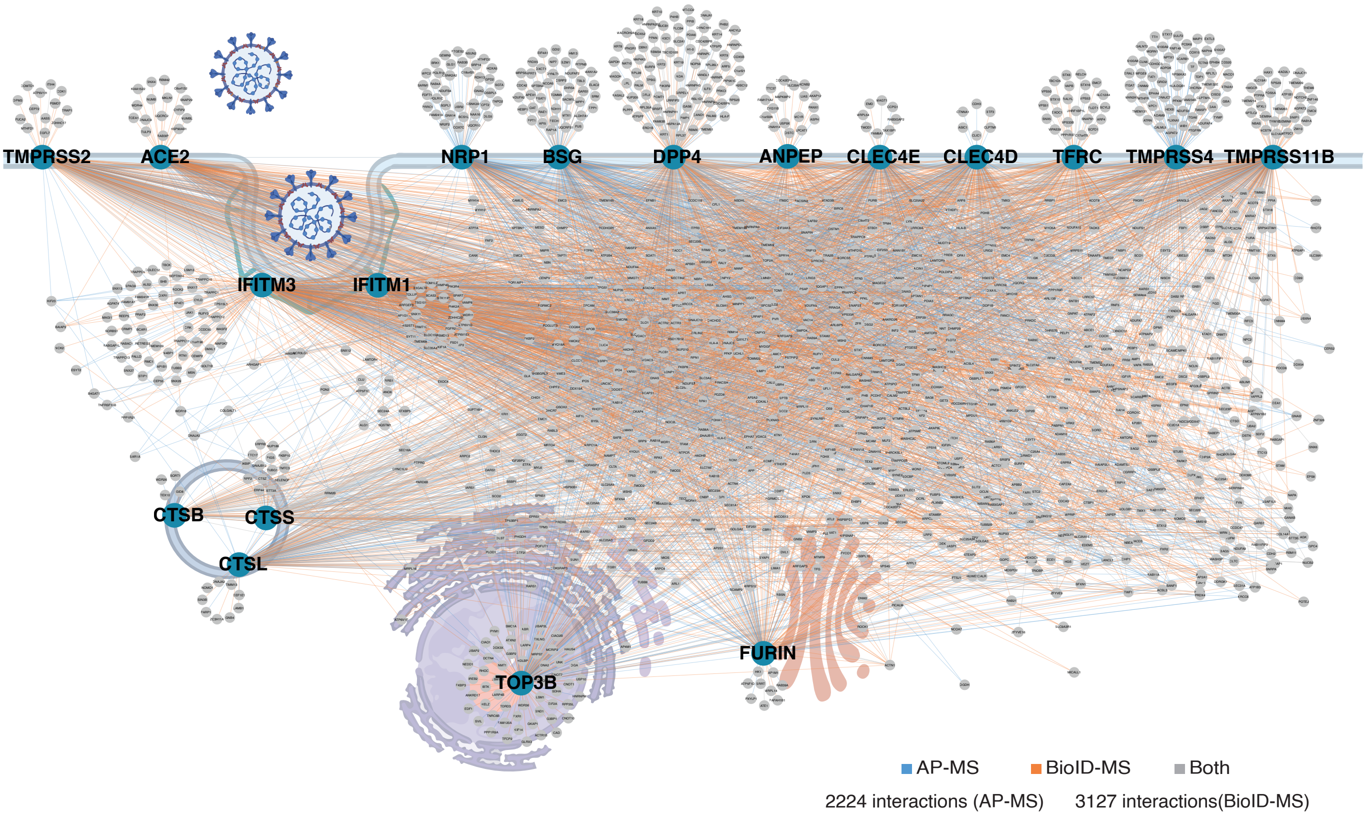

Appendix Figure S3. Human receptor protein-protein interaction map.
Nodes of different colors and sizes represent the bait (cyan) and prey (grey). Connections are colored based on the detection approach with AP-MS: cyan, BioID-MS: orange or both: gray.

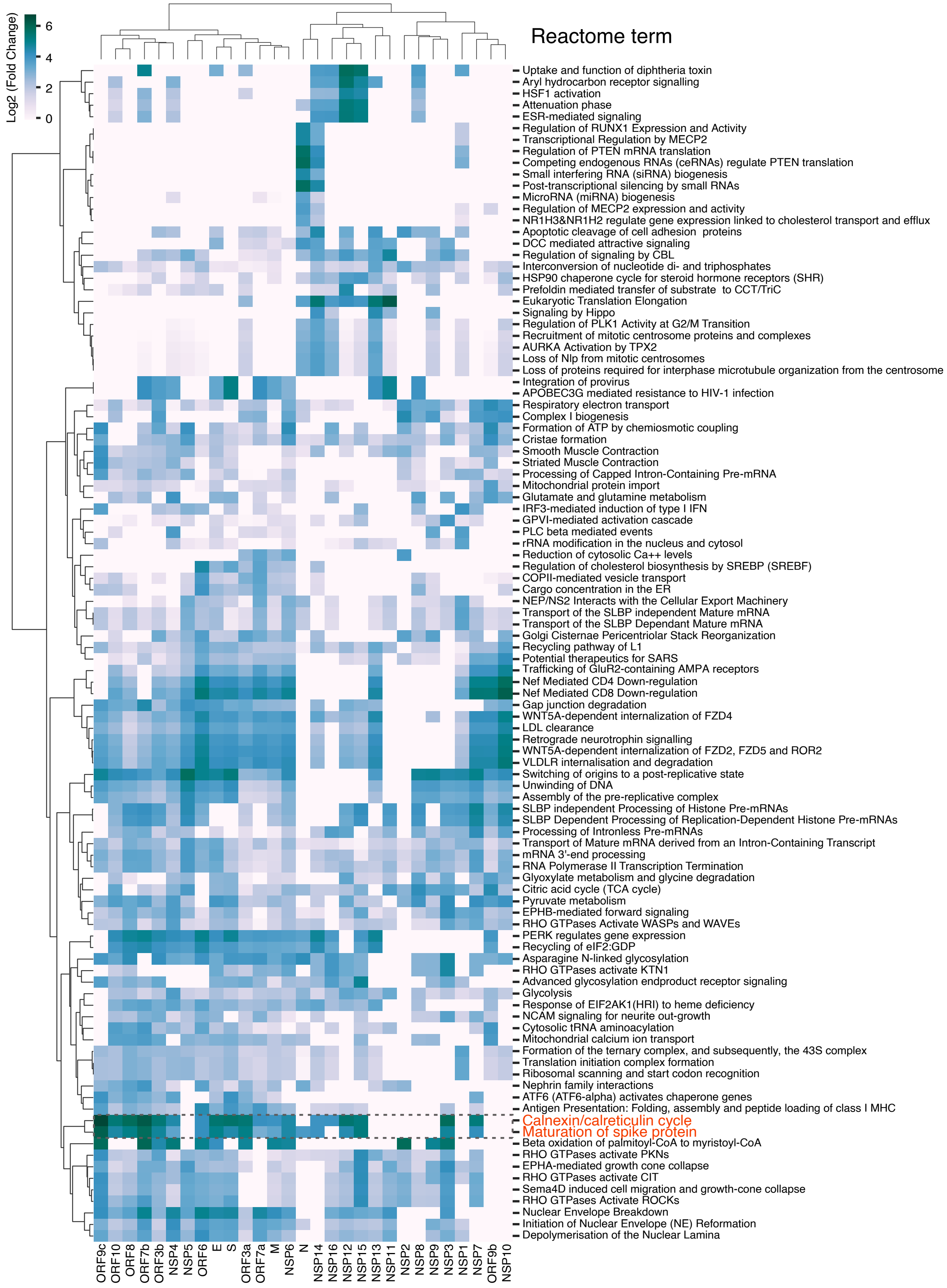

Appendix Figure S4. Enriched Reactome pathways of prey proteins of the virus-host interactome. Analysis of enriched functional terms from Reactome within preys detected by 29 viral baits.

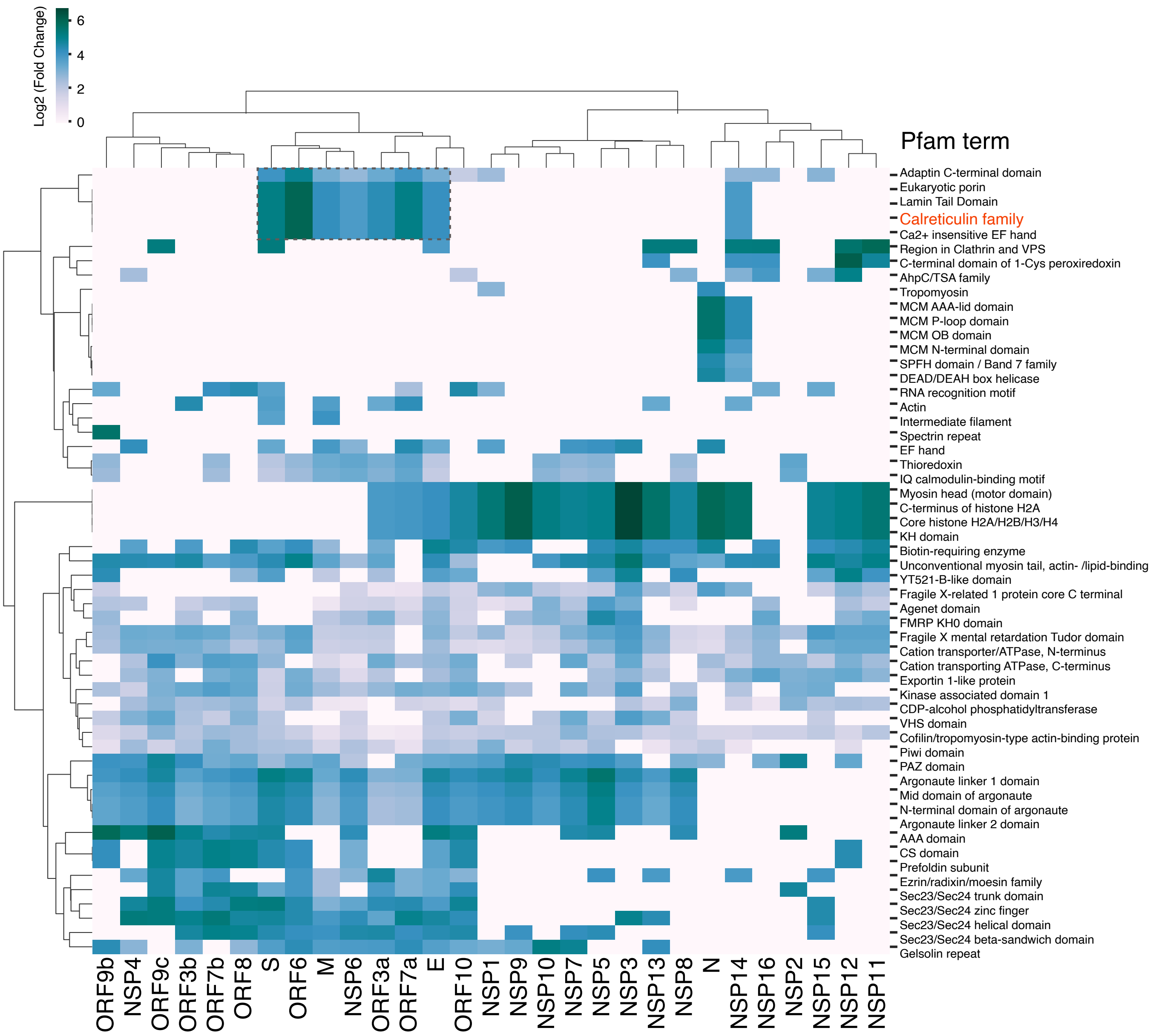

Appendix Figure S5. Enriched Pfam terms of prey proteins of the virus-host interactome.
Analysis of enriched functional terms from Pfam within preys detected by 29 viral baits

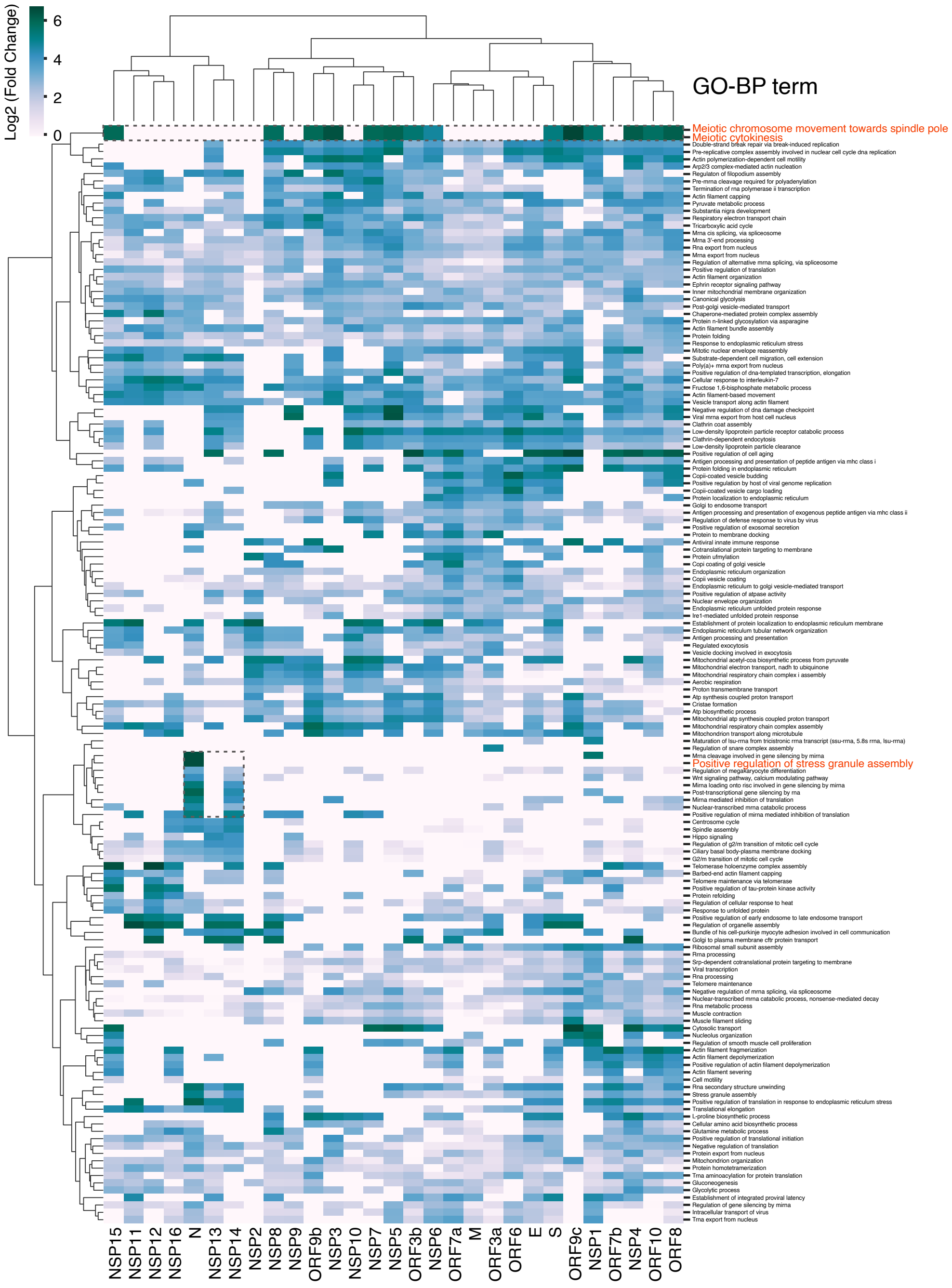

Appendix Figure S6. Enriched GO biological process terms of prey proteins of the virus-host interactome.

Analysis of enriched functional terms from GO biological processes within preys detected by 29 viral baits.

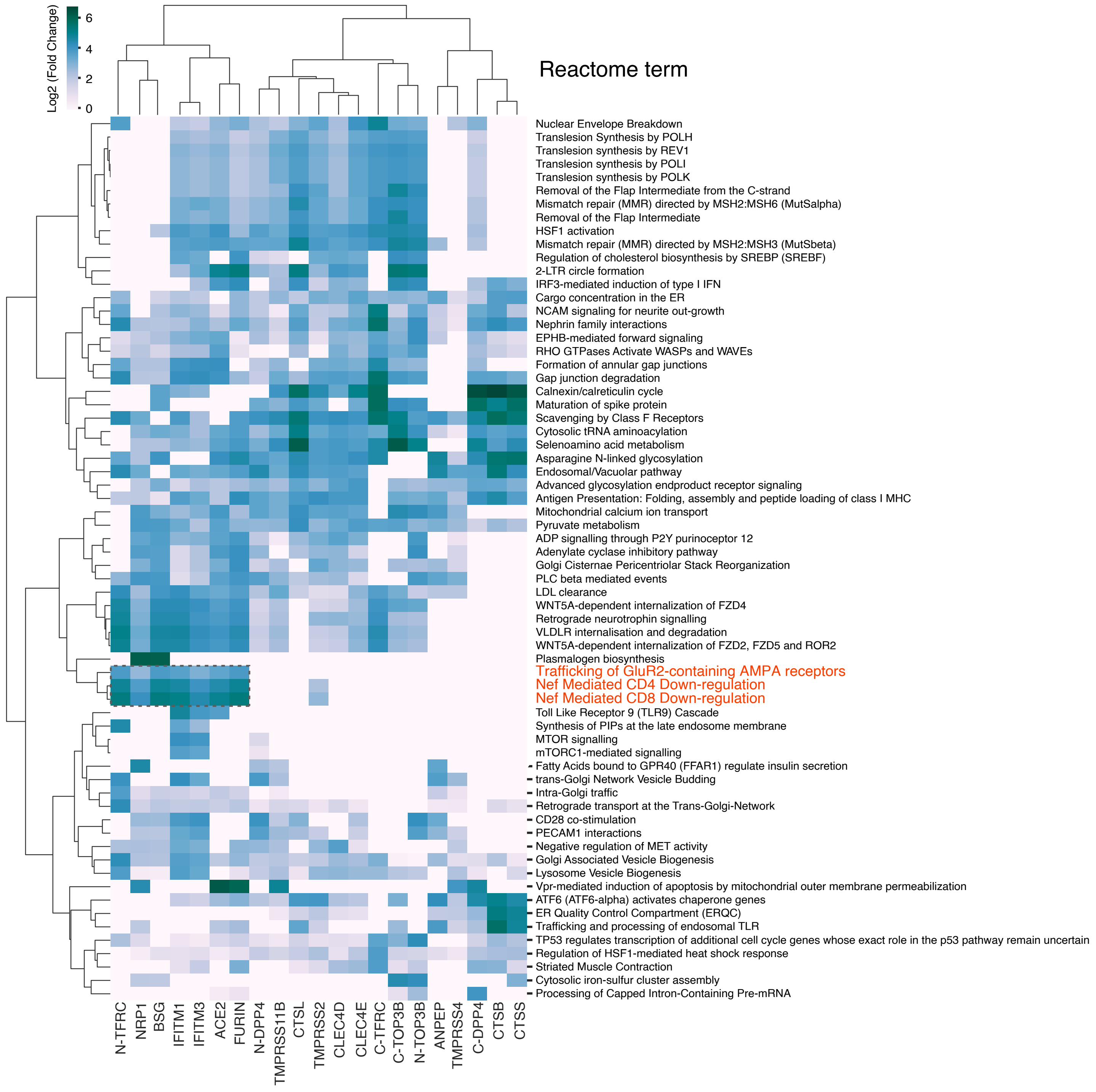

Appendix Figure S7. Enriched Reactome pathways of prey proteins of the host receptor interactome.

Analysis of enriched functional terms from Reactome within preys detected by 18 host baits.

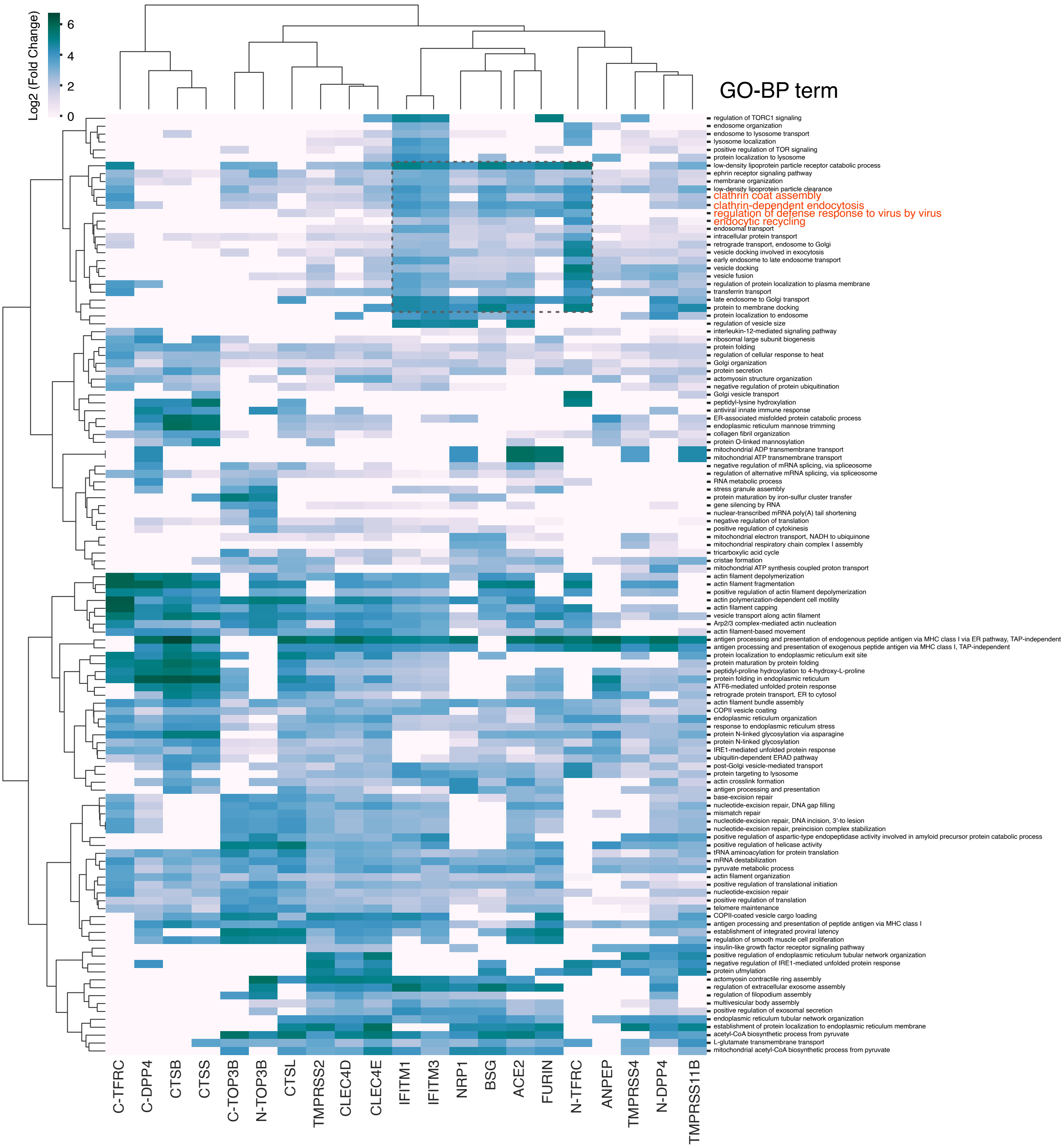

Appendix Figure S8. Enriched GO biological process terms of prey proteins of the host receptor interactome.

Analysis of enriched functional terms from GO biological process within preys detected by 18 host baits.

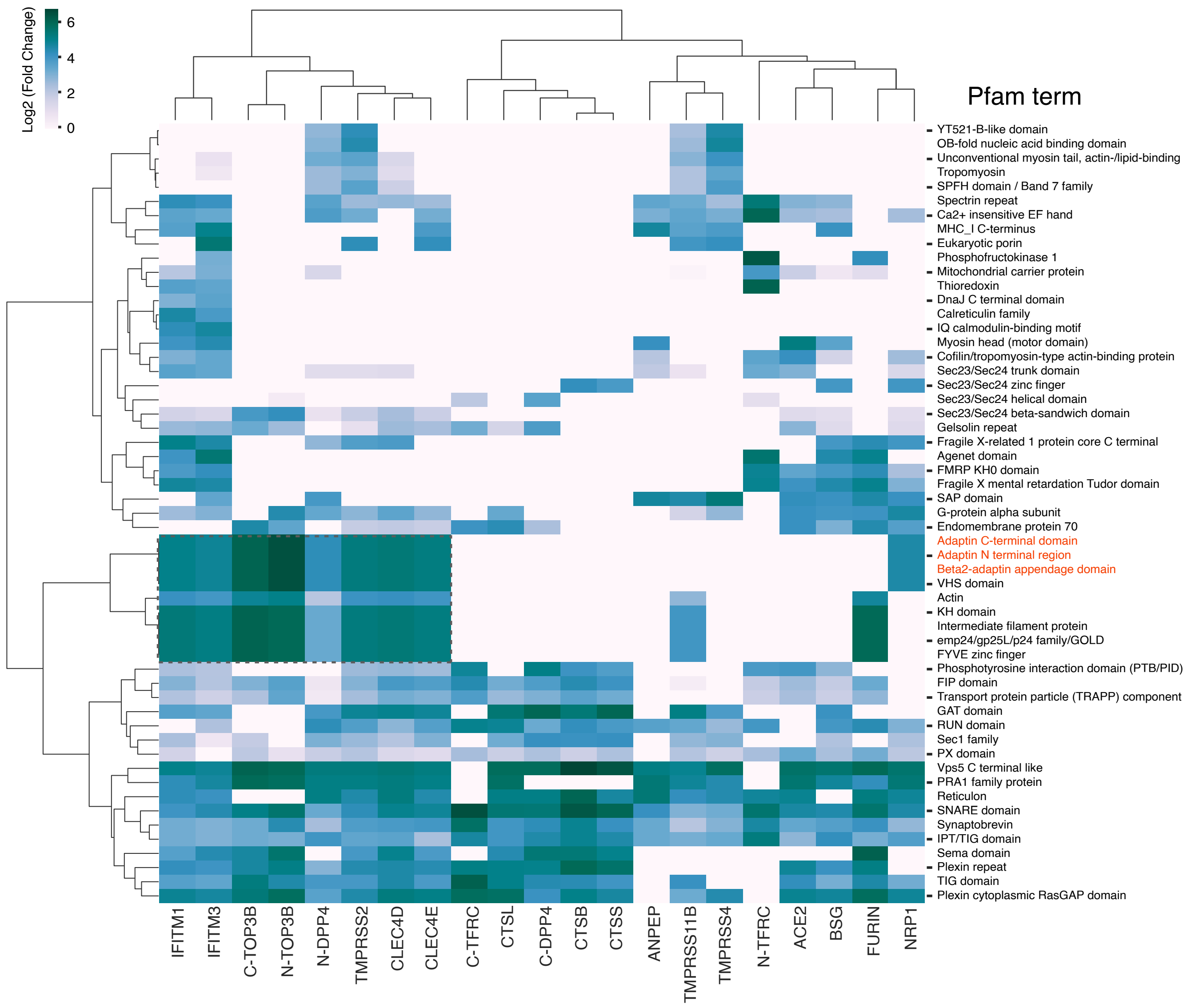

Appendix Figure S9. Enriched Pfam terms of prey proteins of the host receptor interactome. Analysis of enriched functional terms from Pfam within preys detected by 18 host baits.

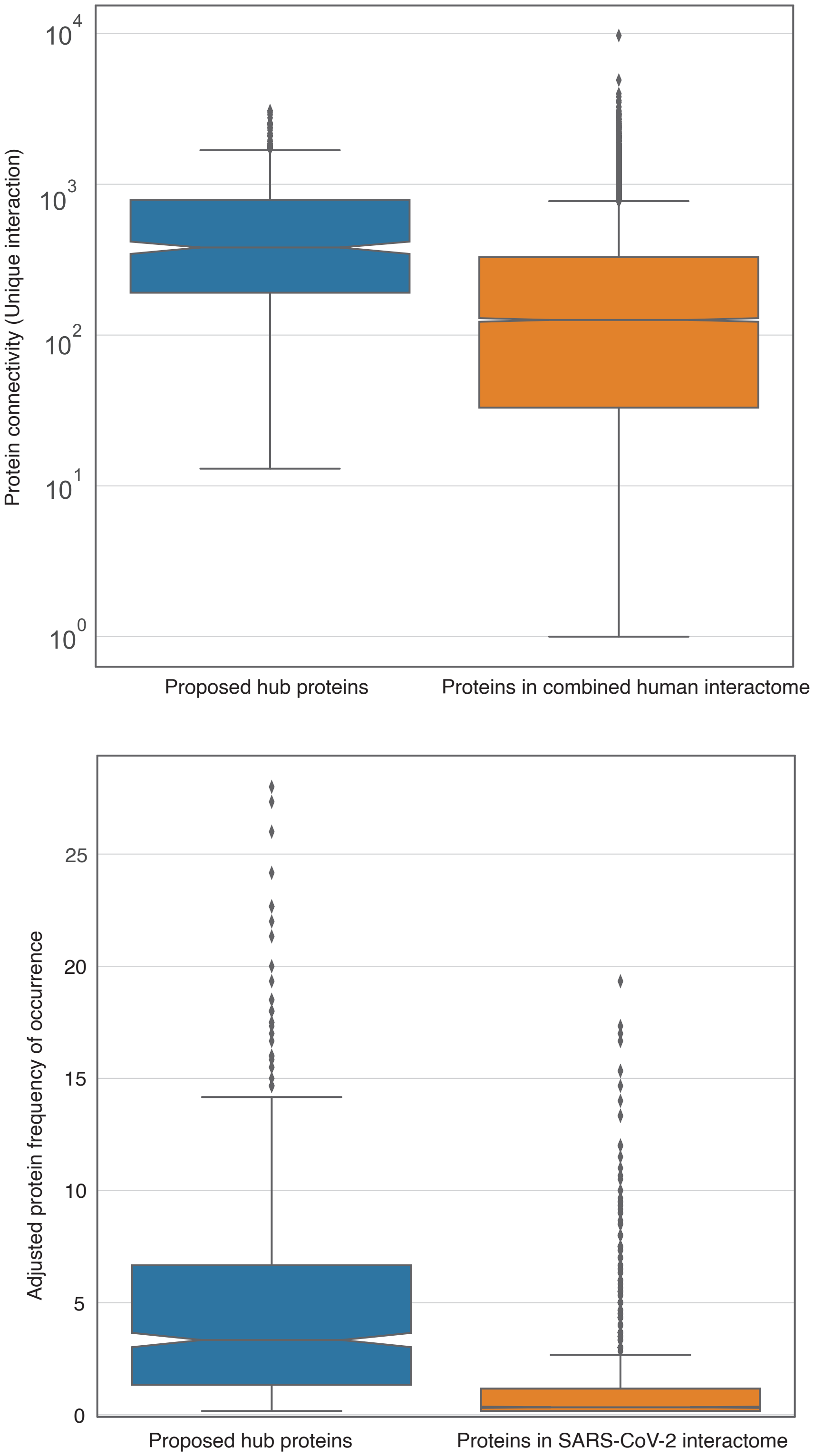

Appendix Figure S10. Distribution of frequencies of hub proteins.
A Notched box plots comparing the distribution of protein connectivity between groups of proposed hub proteins and the other proteins present in combined human interactome.
B Notched box plots comparing the distribution of adjusted frequency metrics between groups of proposed hub proteins and the other prey obtained in the combined SARS-CoV-2 interactome (3 AP and 3 BioID datasets).

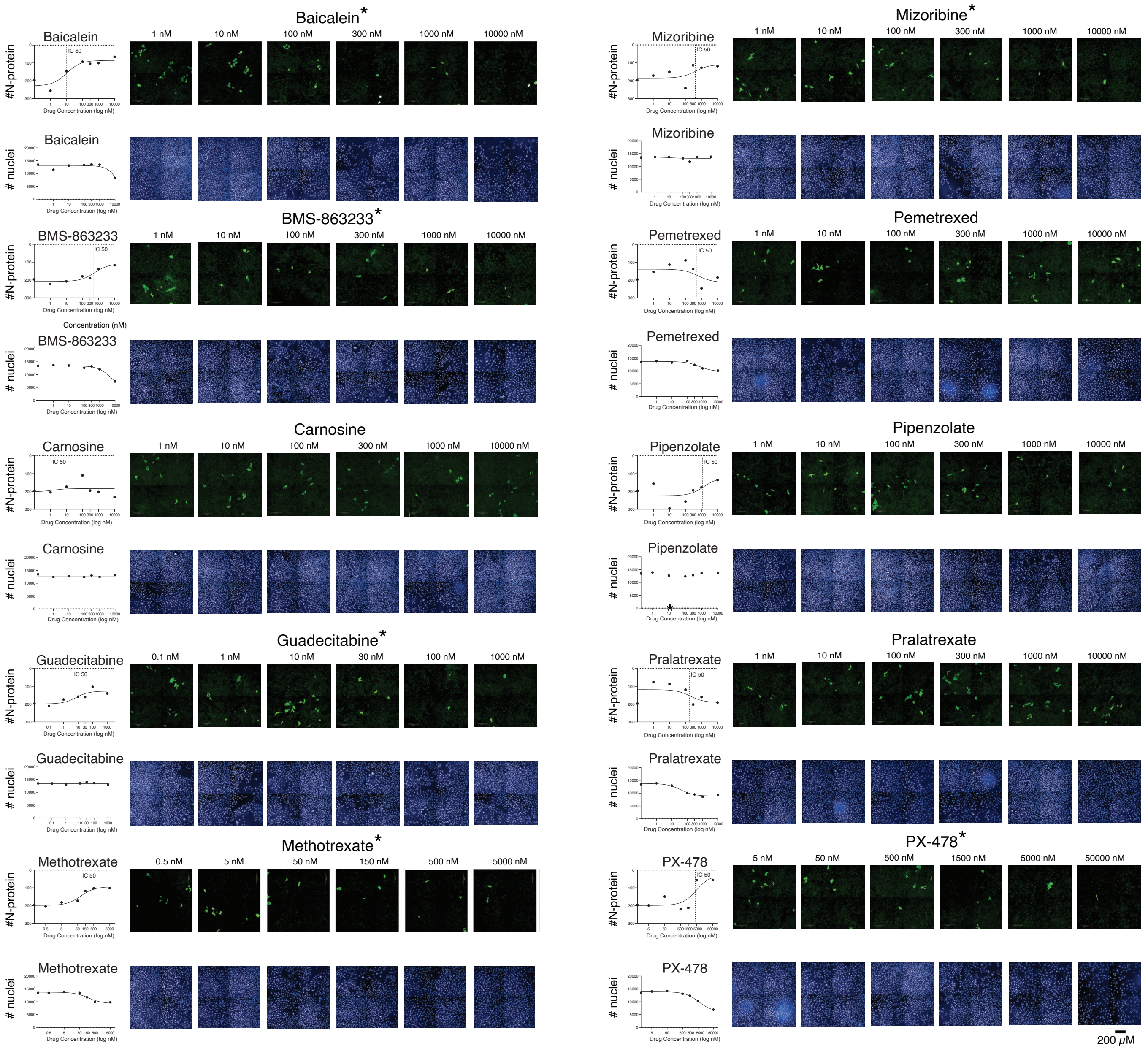

Appendix Figure S11. The antiviral activity of proposed repurposing candidates in the image-based drug screening of SARS-CoV-2.

Drug response curves are calculated based on the amount of N protein positive cells (above) and count of nuclei in six different concentrations of each drug (below). IC50 values are marked to the graphs. Immunofluorescence microscopy images representing SARS-CoV-2 infected cells based on N protein immunostaining (N protein, green; nuclei, blue) in the presence of the drug are shown. Drugs with potential antiviral effects are marked (*). Scale bars 200 μm.

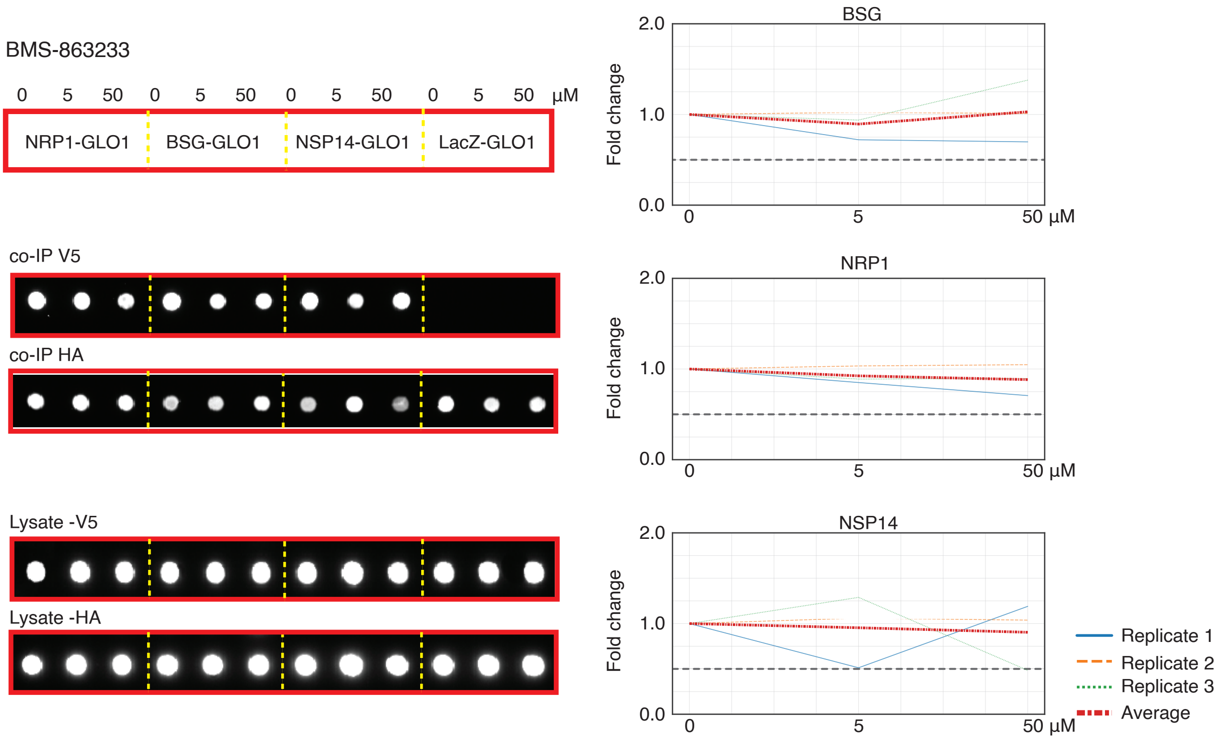

Appendix Figure S12. The presence of BMS-863233 does not affect interactions formed with GLO1.

Data that represent the mean of 3 replicates are highlighted in red.

A fold change > 0.5 is considered significant.
