# Supplementary material for: SARS‐CoV‐2–host proteome interactions for antiviral drug discovery
Source: Mol Syst Biol. 2021 Oct 28;17(11):e10396. doi: 10.15252/msb.202110396 (PMC8552907; doi:10.15252/msb.202110396)
Supplement: Supplementary file 3 — Expanded View Figures PDF [file MSB-17-e10396-s009.pdf]

Expanded View Figures

Figure EV1. Defining the host–receptor interaction landscape.

A Number of known interactions related to 18 host baits from each of the databases or publications used for constructing the known interaction database.
B Number of previously identified interactions detected in one or more of the databases used.
C Number of known interactions of each human receptor bait.
D The number of high-confidence interactions (HCIs) identified by host baits using AP-MS or BioID-MS methods.
E Human bait protein expression patterns vary greatly in different tissues.

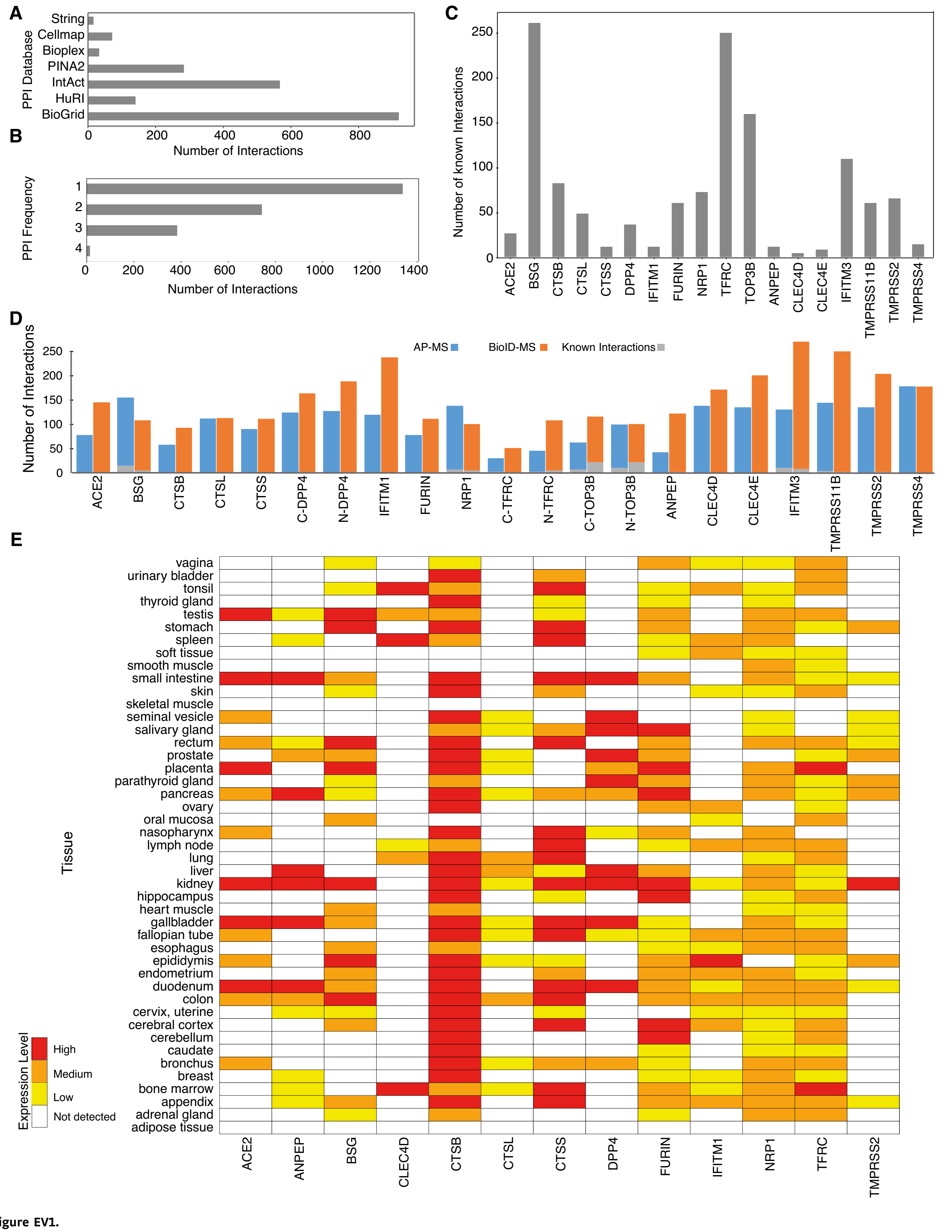

Figure EV1.

 © 2021 The Authors

Figure EV2. Defining the virus–host interaction landscape.

A, B Venn diagrams show high-confidence bait–prey interaction pair overlap of three virus–host PPI studies using either AP-MS (A) or BioID-MS (B). Duplicate PPIs were removed from each study.
C The number of HCIs identified by viral baits using AP-MS or BioID-MS.
D, E Frequency of prey proteins detected by baits using AP-MS (D) or BioID-MS (E).
F, G Venn diagrams show high-confidence interactor overlap of three virus–host PPI studies using either AP-MS (F) or BioID-MS (G).

© 2021 The Authors

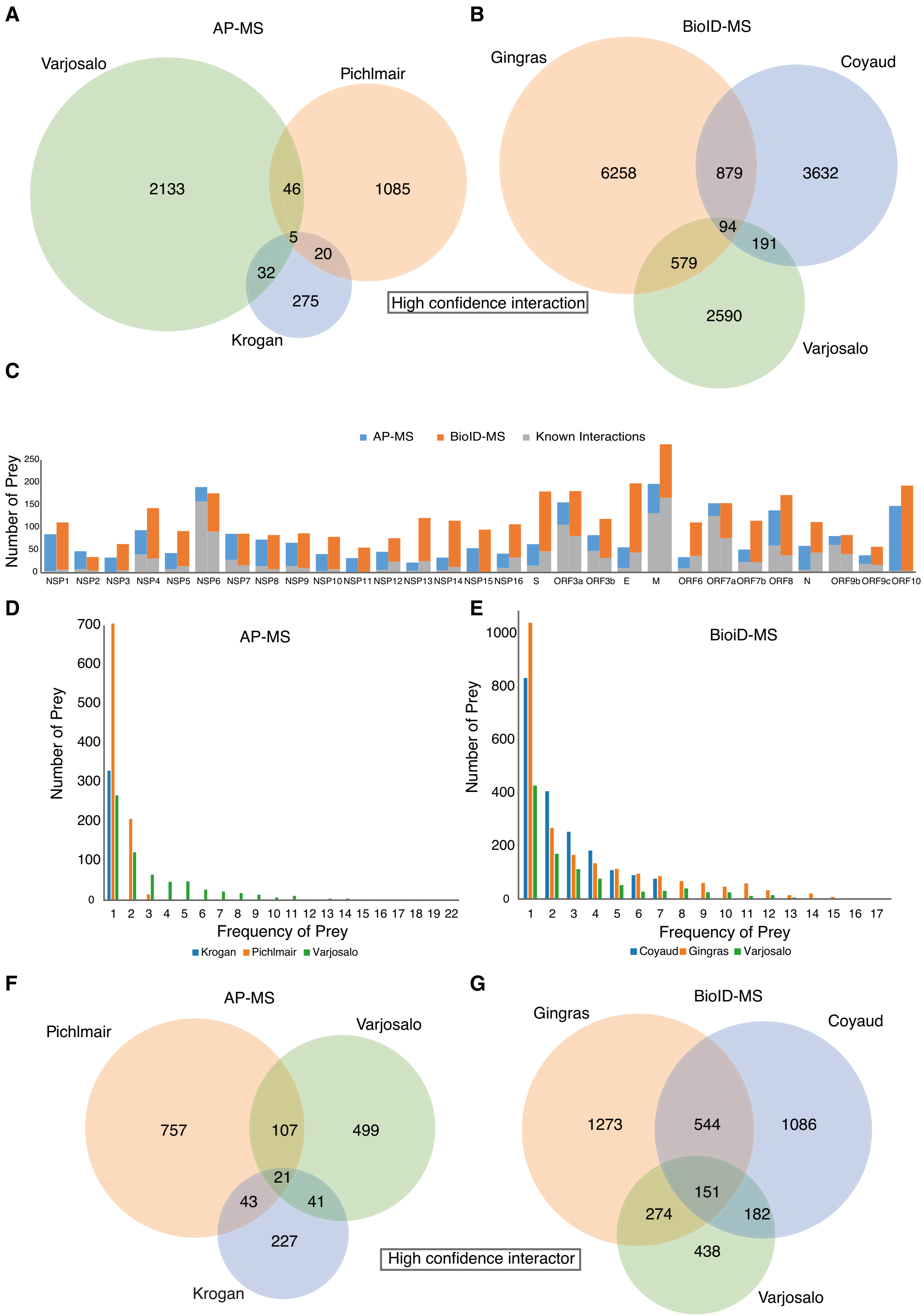

Figure EV2.

© 2021 The Authors

Figure EV3. Validation of interaction data by Co-IP and dot blotting.

A, B Proteomics interaction data were evaluated by Co-IP and dot blotting, related to Dataset EV6. In total, 190 randomly selected interaction pairs from both virus–host interactome (A) and receptor interactome (B) were confirmed by Co-IP. The prey protein tagged with a Strep-HA tag and bait protein tagged with V5 were co-expressed in HEK293 cells. The Strep-HA-tagged GFP with V5-tagged LacZ constructs was used as the negative control. Strep-HA-tagged proteins were immunoprecipitated with the Strep-Tactin Sepharose resin, and then, the immunoprecipitated complexes were analyzed by dot blotting with anti-V5 antibody and anti-HA antibody, respectively. Five percent of the total cell lysate was used as the input loading control.

© 2021 The Authors

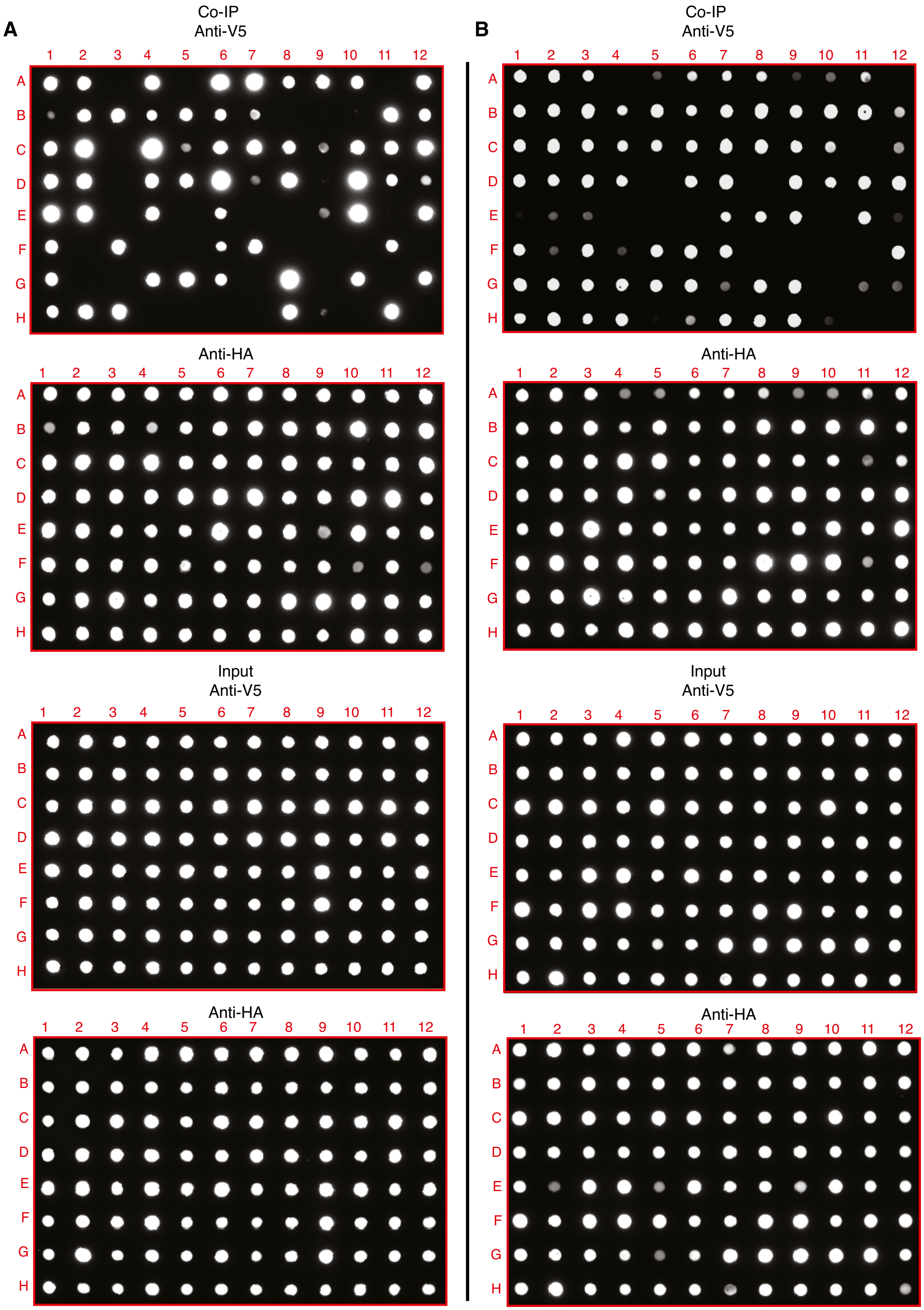

Figure EV3.

 © 2021 The Authors

Figure EV4. Unique subcellular structure upon overexpression of NSP3.

A Heatmap showing the molecular context of 29 viral bait proteins based on the annotation score of the MS-microscopy system. The color scheme is shown in the bottom part of the panel. The right-hand panel shows the architecture of the host cytoskeleton in NSP3-transfected U2-OS cells. F-actin was detected with phalloidin (Scale bar: 10 μm).

B The heatmap shows average spectral counts for prey detected by MAC-tagged NSP3.

C The summary of GO molecular function associated with high-confidence interactors of NSP3 was visualized by treemap. The size of each box was correlated to the *P*-value of the GO term.

D Upregulated pathways in phosphoproteomic data of NSP3 were revealed by KEGG pathway analysis.

© 2021 The Authors

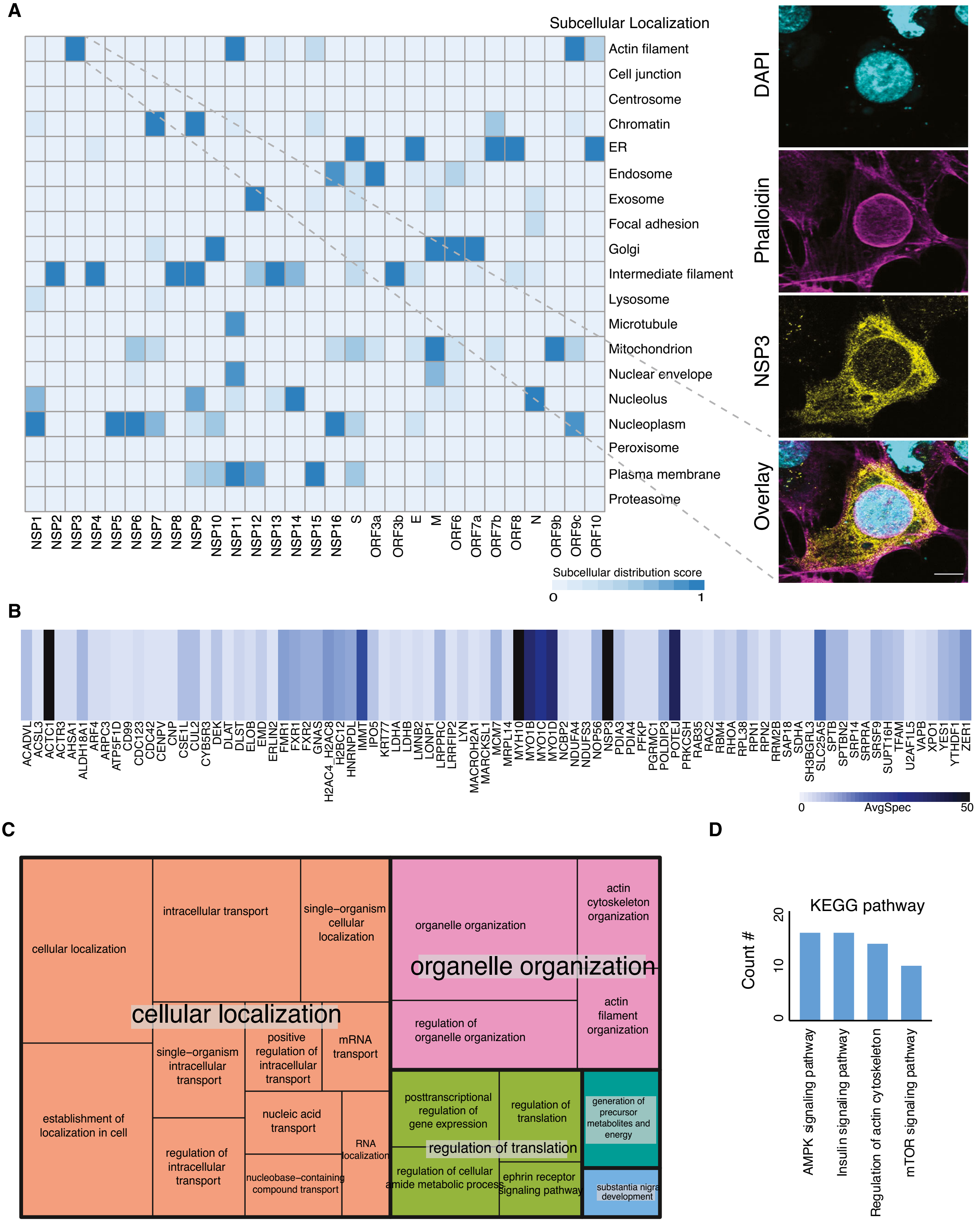

Figure EV4.

 © 2021 The Authors

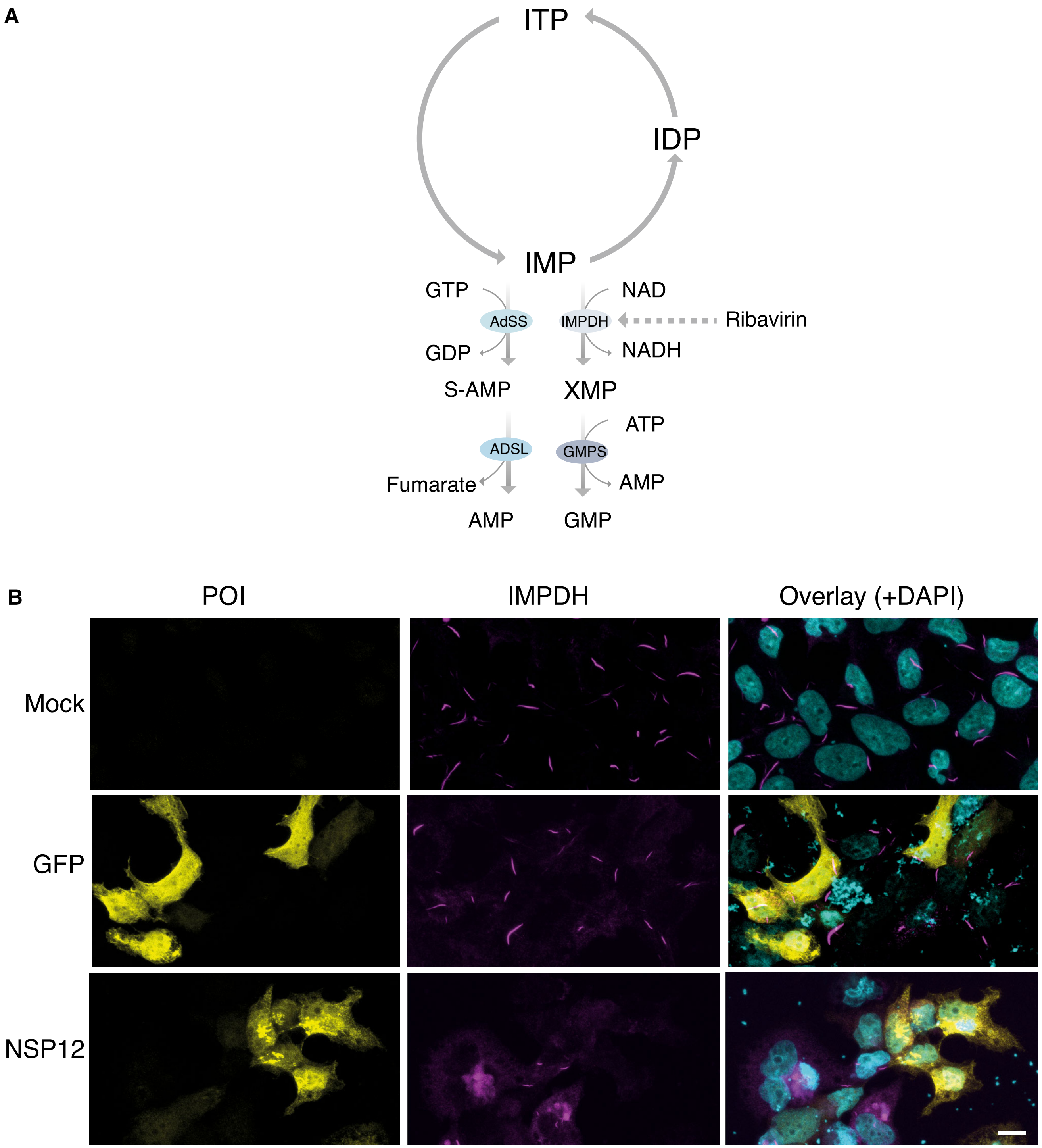

Figure EV5. Overexpression of viral ORFs suppresses ribavirin-induced "rod and ring" structures.

A Conversion of ITP to GMP and AMP, and the nucleotide salvage pathway. Ribavirin targets IMPDH to inhibit guanine synthesis.

B Representative immunofluorescence images showing the morphology of RR structures under ribavirin induction in untransfected and GFP-transfected cells. Transfection of viral ORF (NSP12) expression vectors suppresses ribavirin-induced RR structures. Scale bar: 10 µm.

© 2021 The Authors
